# Validation of a Cellular Imaging‐Based Method as a Potential Biomarker for SPG4 Hereditary Spastic Paraplegia

**DOI:** 10.1002/acn3.70482

**Published:** 2026-09-02

**Authors:** Gaia Fattorini, Valerio Licursi, Gianmarco Dalla Zanna, Fabio Dal Canto, Melissa Barghigiani, Nunzio Setola, Salvatore Rossi, Antonio Funcis, Filippo M. Santorelli, Gabriella Silvestri, Carlo Casali, Francesca Sardina, Cinzia Rinaldo

**Affiliations:** ^1^ IBPM‐CNR Rome Italy; ^2^ Department of Neurosciences Università Cattolica del Sacro Cuore Rome Italy; ^3^ Molecular Medicine, IRCCS Fondazione Stella Maris Pisa Italy; ^4^ Department of Neurosciences, Sensory Organs and Thorax Fondazione Policlinico Universitario A. Gemelli IRCCS Rome Italy; ^5^ Sapienza University of Rome Latina Italy

**Keywords:** biomarker, cellular imaging, hereditary spastic paraplegia, microtubule, SPG4, target‐engagement

## Abstract

**Background:**

Hereditary Spastic Paraplegia (HSP) comprises a group of rare genetic diseases characterized by length‐dependent axonal degeneration of the corticospinal tracts and dorsal columns, whose main clinical feature is spastic gait. Pathogenic variants in the *SPG4* gene cause Spastic Paraplegia Type 4 (SPG4‐HSP), the most common form of HSP. *SPG4/SPAST* encodes spastin, a protein involved in microtubule regulation and lipid droplet behavior. SPG4‐HSP shows extreme heterogeneity in both clinical manifestations and genetics. Although no cure is currently available, several strategies aimed at restoring spastin levels are emerging; however, SPG4‐HSP still lacks accessible cellular readouts for clinical studies. This study evaluates a cell‐imaging approach that quantifies the distance between nucleus and cell centroid (dcnc) in peripheral blood mononuclear cells (PBMCs) in a genetically and clinically heterogeneous SPG4‐HSP cohort.

**Methods:**

PBMCs from 48 molecularly confirmed SPG4‐HSP patients and 21 healthy controls (HC) were analyzed by automated cell imaging. Dcnc and additional cytoskeletal and lipid droplet‐related parameters were measured. Patient‐level discrimination was assessed via cross‐validated classification; correlations with molecular and clinical features were explored.

**Results:**

At the patient level, dcnc distinguishes SPG4‐HSP from HC, independently of mutation type and disease severity, supporting robust cross‐validated classification and inverse correlation with spastin protein levels.

**Conclusion:**

Dcnc is a robust, non‐invasive, and mutation‐independent cellular candidate biomarker for SPG4‐HSP. It also offers preliminary evidence consistent with spastin target engagement supporting evaluation in future clinical trials.

**Abbreviations**
AE: age at examination
AO: age at onset
AUC: area under the curve
CI: confidence interval
dcnc: distance between nucleus and cell centroid
DMSO: dimethyl sulfoxide
DP: disease progression
ES: effect size
HC: healthy controls
HSP: hereditary spastic paraplegia
LCLs: lymphoblastoid cell lines
LD: lipid droplets
MT: microtubules
NA: numerical aperture
PBMCs: peripheral blood mononuclear cells
PHA: phytohaemagglutinin
ROC: receiver operating characteristic
SPRS: Spastic Paraplegia Rating Scale

## 1 Background

Hereditary spastic paraplegia (HSP) is a group of rare, hereditary neurodegenerative diseases characterized by extreme heterogeneity in both clinical manifestations and genetic background [1, 2, 3]. HSP type 4 (SPG4‐HSP) is the most prevalent pure form, inherited in an autosomal dominant manner. SPG4‐HSP is caused by mutations in the *SPG4/SPAST* gene, which encodes spastin, a microtubule (MT) severing ATPase involved in cytoskeletal rearrangements associated with membrane and MT dynamics, and in lipid droplet (LD) regulation [4, 5, 6, 7, 8, 9]. Spastin has four isoforms generated by alternative translational initiation sites and splicing [10, 11]. All isoforms share key functional domains, including the AAA ATPase domain, the MT‐binding domain (MTBD), and the MIT domain, while the M1 isoform contains an additional N‐terminal hydrophobic region implicated in membrane interactions.

SPG4‐HSP exhibits marked phenotypic variability. The age at onset (AO) ranges from birth to the seventh decade, and disease severity is highly variable, unpredictable even among individuals carrying the same variant within the same family [3, 12]. Disease progression (DP) is also heterogeneous [13, 14, 15], and typically evaluated using clinical outcome measures. Advanced neuroimaging measures and other candidate molecular biomarkers, such as serum neurofilament light chain (sNfl) are being explored to complement clinical assessment [16, 17, 18], but reliable, objective and easily accessible biomarkers remain lacking.

More than 250 different pathogenic variants of the SPG4 gene have been reported, including frameshift, nonsense, insertions, deletions, and missense mutations, which mainly cluster in the ATPase activity domain [13, 14]. To date, no effective disease‐modifying therapies are available, but approaches based on drugs counteracting dysfunctional mechanisms or spastin‐enhancing treatments are emerging [19, 20, 21, 22, 23, 24]. Therefore, there is a need to develop easily accessible biomarkers that can be used to predict spastin levels and/or functions and facilitate monitoring of treatment efficacy.

We have recently developed a cell imaging‐based method that can distinguish SPG4‐HSP PBMCs from those from HC and other HSP subtypes by measuring the distance between the nucleus and the cell centroid (dcnc) [22]. We showed that an increase in the dcnc measure is indicative of alterations in the MT cytoskeleton organization of PBMCs and lymphoblastoid cell lines (LCLs) from a small subset of *SPG4* patients, and can be induced by reducing spastin protein levels or inhibiting its activity in cells from HC. The alterations in the MT cytoskeleton organization of *SPG4* cells are reversible and can be restored by increasing spastin levels [22, 23], supporting the functional link with the disease and the possibility that dcnc is used to monitor the effects of spastin‐enhancing treatments in non‐neuronal cells.

In this study, we evaluated the clinical utility of the dcnc, alone or combined with additional cellular parameters, in a large, clinically and genetically heterogeneous cohort of SPG4‐HSP patients.

## 2 Methods and Materials

### 2.1 Study Cohort and Ethics Statement

Peripheral blood mononuclear cells (PBMCs) were obtained from 48 genetically confirmed SPG4‐HSP patients, recruited across three Italian centers. The cohort was selected to encompass a broad spectrum of clinical severity and mutation types. PBMCs from 21 HC were also collected.

The study was approved by the Ethics Committee of ASL Roma 2 (protocol number 0074975/2020) and the Tuscany regional CEPR (IRB reference codes CEPR 102/2020 and CEPR 03/2024). Written informed consent was obtained from all participants. Clinical evaluations and genetic counseling were performed as part of standard care. Data analysis was performed anonymously.

### 2.2 PBMC Isolation and Cell Culture

PBMCs were isolated and processed under pre‐specified pre‐analytical conditions, including fixed anticoagulant (ethylenediaminetetraacetic acid), storage temperature (room temperature), and maximum time‐to‐processing prior to isolation (72 h) as in 22. Briefly, PBMCs were isolated from anticoagulant blood samples using Lympholyte‐H (Cedarlane) density gradient centrifugation. Cells were stimulated with 5 μg/mL phytohaemagglutinin (PHA, Thermofisher, 10576015) for 60 h to promote cell cycle entry and cultured in RPMI GlutaMAX (Gibco) supplemented with 10% FBS. All samples were processed and cultured under identical conditions.

### 2.3 Cell Imaging

Cells were seeded onto poly‐L‐lysine coated coverslips, fixed in 4% formaldehyde and then blocked in 5% bovine serum albumin in PBS before the primary Ab (anti‐β‐tubulin‐Cy3 #C4585 1:300, Sigma‐Aldrich) was applied, as in 22. Bodipy 493/503 (D3922, Life technologies) and Hoechst (Sigma‐Aldrich) were used to mark LD and DNA, respectively. Cells were examined under an inverted microscope (Eclipse Ti, Nikon) using a Clara camera (Andor technology) and a 60× oil objective (NA = 1.4). Images for each sample were taken in parallel under identical microscope settings by NIS‐Elements H.C. 5.11 using the JOBS module for automated acquisitions. Automated image analysis was performed using custom CellProfiler pipelines [22], extracting the following parameters for each cell: (1) intensity of β‐tubulin calculated as the mean pixel intensity within a selected region of interest (ROI); (2) form factor is calculated as 4π × area/perimeter2 of β‐tubulin ROI, (3) dcnc calculated as the length of the line segment joining two points (i.e., the nucleus and cell centroids, set to the Hoechst and β‐tubulin ROIs, respectively). (4) LD number, count of Bodipy‐positive spots within β‐tubulin ROI; (5) LD intensity calculated as the mean pixel intensity of Bodipy signal within β‐tubulin ROI. For LD analysis, segmentation was performed using histogram‐based thresholding algorithms included in the CellProfiler pipeline. The CellProfiler pipeline used in this study is provided as File S1.

### 2.4 Spastin Protein Level Quantification and Familial Subset

PBMCs from seven carriers of the *SPG4* c.1215_1219delTATA variant belonging to three unrelated families were analyzed. Spastin protein levels were assessed by Western blot: total cell extracts were prepared in RIPA buffer and separated by SDS polyacrylamide gel. Immunoreactivity was determined using ECL Prime (Amersham); image acquisition and densitometry analysis was performed with Image Lab software (Bio‐Rad). The following antibodies (Abs) were employed: anti‐spastin (clone sp6c6 #sc‐81624 1:1000; Santa Cruz Biotechnology), and anti‐mouse‐HRP (#7076 Cell Signaling Technology).

### 2.5 Statistical Analysis

All inferential analyses were conducted at the patient level using the per‐subject median. Data normality was assessed by using the Shapiro–Wilk test. For non‐normally distributed data, Mann–Whitney tests were used instead of unpaired Student's *t*‐test. Statistical significance was set as follows: ****p < 0.00001, ***p < 0.0001, **p < 0.001, and *p < 0.05. Kolmogorov–Smirnov tests were used to explore distributional differences between groups when comparisons were performed at single‐cell level and effect size (Cohen's *d*) was also calculated to provide a standardized measure of the magnitude of group‐level differences, that is independent of sample size. 95% confidence intervals (CI) were reported. Pearson correlations between median dcnc and normalized spastin levels were computed at the subject level. Receiver Operating Characteristic (ROC) curve and Area Under the Curve (AUC) analyses was calculated in GraphPad Prism 8 with 95% confidence intervals using the Wilson‐Brown method.

## 3 Results

### 3.1 Dcnc‐Based Cell Imaging Analysis in a Cohort of SPG4‐HSP Patients

PBMCs from 48 genetically confirmed *SPG4* patients were selected (Table 1) to reflect the clinical and molecular heterogeneity of the disease [13, 14]. The mean AO, measured as the time to first manifestation of symptoms, was 28.3 ± 18.4 years, and 62.5% of patients were male. The mean disease duration (calculated as the time from AO to age at examination, AE) was 19.6 ± 15.8 years. Disease severity ranged from 0 to 34 (mean 15 ± 11.8 SD), measured by the Spastic Paraplegia Rating Scale (SPRS), which is a reliable clinical instrument for HSP severity/progression widely endorsed in clinical practice [25]. Notably, nine patients (18.7%) had an SPRS score of 0 at the time of examination. They exhibit subtle neurological signs but no clinically relevant symptoms of HSP, and were therefore classified as asymptomatic, non‐manifesting carriers. A total of 26 distinct *SPG4* variants were represented in the cohort, comprising missense, nonsense, frameshift, splicing, duplication and deletions (Figure S1A,B and Table 1).

**TABLE 1:** Molecular and clinical features of the SPG4‐HSP cohort.

*SPG4* variant	Amino acid change	Gender	Age at onset	Age at examination	SPRS	Disease progression
c.1215_1219delTATAA	p.N405KfsX36	F	20	52	28	0.88
c.1215_1219delTATAA	p.N405KfsX36	F	Asympt	52	0	—
c.1215_1219delTATAA	p.N405KfsX36	F	5	52	13	0.28
c.1304C>T	p.P435L	F	30	49	24	1.26
c.546G>T	p.G147X	M	50	51	26	26.00
c.546G>T	p.G147X	M	3	10	5	0.71
c.1216A>G	p.I406V	M	29	60	34	1.10
c.587‐?_1851+?del (ex 4‐17)	p.E196_V616del	M	44	58	17	1.21
c.587‐?_1851+?del (ex 4‐17)	p.E196_V616del	M	33	34	1	1.00
c.1‐?_415+?del (ex1)	p.M1_A139del	M	13	56	30	0.70
c.322_334delGTGCCGGGCGGCG	p.V108RfsX49	M	47	57	31	3.10
c.683‐2A>G	Undefined splicing effect	M	40	51	12	1.09
c.1378C>T	p.R460C	M	55	63	10	1.25
c.1378C>T	p.R460C	M	50	66	29	1.81
c.1537‐8T>G	Undefined splicing effect	M	22	34	12	1.00
c.1553T>C	p.L518P	M	50	55	28	5.60
c.1553T>C	p.L518P	M	21	23	10	5.00
c.1094C>G	p.P365R	M	20	73	30	0.57
c.1094C>G	p.P365R	F	Asympt	70	0	—
c.1553T>C	p.L518P	M	45	60	20	1.33
c.1553T>C	p.L518P	F	0	30	25	0.83
c.870G>A	p.K290K	F	30	58	20	0.71
c.1‐?_415+?del (ex1)	p.M1_A139del	F	41	55	10	0.71
c.1512_1515dup	p.V506IfsX	F	56	60	20	5.00
c.1215_1219delTATAA	p.N405KfsX36	M	43	65	30	1.36
c.1536G>C	p.E512D	M	Asympt	49	0	—
c.1536G>C	p.E512D	M	1	22	4	0.19
c.1246‐?_1728+?del (ex 10‐16)	p.V416_E576del	F	48	63	28.5	1.90
c.1246‐?_1728+?del (ex 10‐16)	p.V416_E576del	M	27	32	1.5	0.30
c.1729?_1851+?del (ex 17)	p.M577_V616del	M	43	51	14.5	1.81
c.89C>T	p.A30V	F	Asympt	51	0	—
c.1496G>A	p.R499H	F	2	4	7	3.50
c.1728+1G>A	Undefined splicing effect	M	2	3	5	5.00
c.1728+1G>A	Undefined splicing effect	F	Asympt	47	0	—
c.1378C>T	p.R460C	M	2	22	29	1.45
c.1378C>T	p.R460C	F	Asympt	16	0	—
c.1378C>T	p.R460C	F	31	46	16	1.07
c.864_865dupTC	p.H289LfsX26	M	41	51	17.5	1.75
c.1728+1G>C (c.1853G>C)	p.1_577	M	1	52	33	0.65
c.1728+1G>C (c.1853G>C)	p.1_577	M	30	65	10	0.29
c.1728+1G>C (c.1853G>C)	p.1_577	M	30	65	33	0.94
c.751_752insA	p.T251NfsX14	F	6	65	21	0.36
c.751_752insA	p.T251NfsX14	F	Asympt	35	0	—
c.870G>A	p.K290K	M	13	35	20	0.91
c.231G>A	p.W77X	M	20	53	4	0.12
c.870G>A	p.K290K	F	Asympt	67	0	—
c.1215_1219delTATAA	p.N405KfsX36	M	Asympt	39	0	—
c.1617_1728dup (ex 15‐16)	p.R539_E576dup	M	60	67	11	1.57

PBMCs from the SPG4‐HSP cohort and 21 HC were analyzed by cell imaging to measure dcnc and other parameters related to subcellular compartments affected by spastin dysfunction, such as LD and MT (Figure S1C). Specifically, five key parameters were quantified for each cell: dcnc, form factor, tubulin intensity, LD number (nLD) and LD intensity.

Cells were stained for β‐tubulin (MT), with bodipy (LDs), and hoechst (nuclei), and images were acquired and analyzed using an automated and standardized pipeline [22] implemented with additional parameters, as described in Methods and Materials section.

**FIGURE 1:**
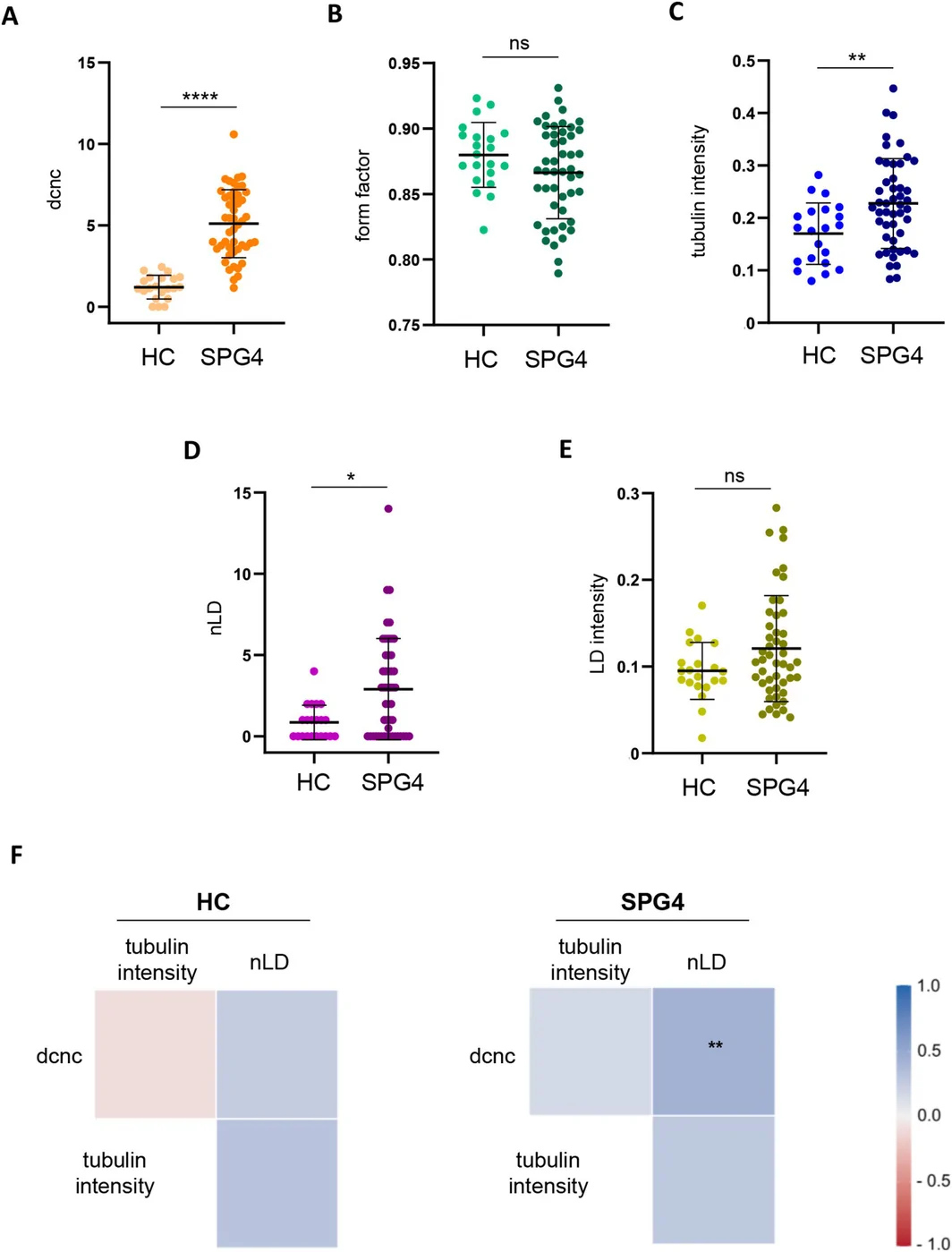
Analysis of PBMCs using dcnc and other parameters. (A–E) Automated analysis of PBMCs derived from HC and *SPG4* patients. At least 500 cells were analyzed per sample. Individual‐level distribution of dcnc, tubulin intensity, nLD, LD intensity, and form factor. Each dot represents the per‐subject median, and the black lines indicate the mean ± SD. ****p < 0.0001; **p < 0.01; *p < 0.05; ns, not statistically significant; *t*‐test. (F) Pearson correlation heatmap among dcnc, tubulin intensity and nLD at individual‐level. Blue indicates positive correlation, white indicates no correlation, and red indicates negative correlation. **p < 0.01.

Single‐cell‐level measurements were obtained and reported as median values for each parameter, followed by comparisons between SPG4 and HC individuals (Figure 1A–E). Significant differences were observed between HC and SPG4 in dcnc, β‐tubulin intensity, and nLD.

Individual single‐cell measurements were further analyzed in an exploratory manner using the Kolmogorov–Smirnov (KS) test to evaluate the discriminatory power of each parameter between SPG4 and HC cells (Figure S2A–E). This analysis indicates that the dcnc is the most effective parameter for distinguishing *SPG4* and HC cells, outperforming the other parameters (KS tests' D: dcnc = 0.42; nLD = 0.24; tubulin intensity = 0.20; LD intensity = 0.17; form factor = 0.07; *p* < 0.001 for all comparisons; Figure S2F). This was further supported by the effect size (Cohen's *d*) analysis with 95% CI (Figure S2G). Dcnc had the highest effect size (*d* = 0.61), followed by nLD (*d* = 0.49) and tubulin intensity (*d* = 0.42) resulting with a medium effect size. LD intensity showed a small‐to‐moderate effect (*d* = 0.31), while form factor had a negligible effect (*d* = −0.13). Collectively, the results demonstrate that the dcnc‐based method is reliable across a highly heterogeneous SPG4‐HSP cohort and represents the most powerful discriminatory cellular parameter among those analyzed.

Next, to explore interdependencies among the three most informative parameters (dcnc, nLD, and tubulin intensity), we performed Pearson correlation analysis. As shown in the heatmap (Figure 1F), a significant positive correlation was observed between dcnc and nLD in SPG4 patients indicating that number of LD increases in parallel with higher dcnc value. No significant associations emerged in the other groups, suggesting that this relationship may represent a pathophysiological feature specific to the SPG4 phenotype.

### 3.2 Dcnc Does Not Correlate With Clinical or Genetic Features of SPG4‐HSP

**FIGURE 2:**
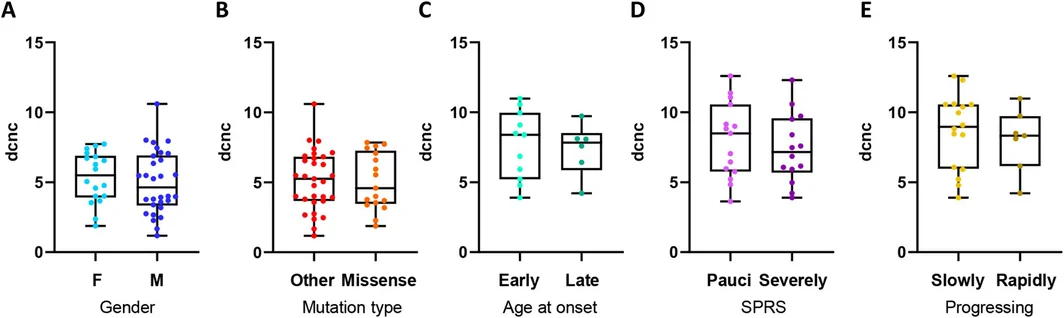
Dcnc across clinical and genetic SPG4‐HSP subgroups. (A–E): Correlation of median dcnc values with gender, molecular, and clinical characteristics of patients. In the box plots, each point represents the median dcnc value for each individual patient across indicated subgroups. In A, gender; in B, mutation type (missense vs. other); in C, AO (early ≤ 15 years vs. late ≥ 50 years); in D, disease severity (SPRS ≤ 5 vs. ≥ 25); in E, progression rate (DP < 1 vs. >2). Statistical analysis was performed using the Mann–Whitney test: A, *p* = 0.51; B, *p* = 0.97; C, *p* = 0.62; D, *p* = 0.66; E, *p* = 0.53.

We assessed whether the dcnc values correlate with clinical or genetic features in SPG4‐HSP patients. For each patient, the median dcnc value was compared across clinical and genetic relevant subgroups (Figure 2). Comparisons were performed according to gender (male, *n* = 30 versus female, *n* = 18; Figure 2A) and mutation type (missense mutations, *n* = 17 versus other, *n* = 31; Figure 2B). The latter subgroup includes nonsense mutations, frameshift mutations, and macro‐deletions resulting in a stop codon, as well as splicing or intronic mutations, including the duplication c.1617_1728dup mutation, which affect a splice intron acceptor site resulting in disrupted RNA splicing and an absent or altered protein product (VCV000574791.1—ClinVar—NCBI).

To explore the relationship between dcnc and clinical parameters, we compared subgroups, clustering patients according to clinically relevant extremes of three key variables. Specifically, for AO the dcnc values of patients with early‐onset (≤ 15 years; *n* = 11) versus late‐onset (≥ 50 years; *n* = 6) were compared (Figure 2C). For disease severity, the dcnc values of asymptomatic or mildly affected patients (paucisymptomatic patients with SPRS score ≤ 5; *n* = 15) versus severe cases (SPRS score ≥ 25; *n* = 14) were compared (Figure 2D). Finally, for yearly DP, the dcnc values of patients showing slow progression rate (DP < 1; *n* = 16) were compared with those of patients showing rapid progression rate (DP > 2; *n* = 7) where DP is SPRS/(AE‐AO) as in 14 (Figure 2E). No statistically significant differences were observed (Figure 2). These findings indicate that the dcnc is not influenced by the analyzed variables within our cohort.

Interestingly, when comparisons were performed among asymptomatic patients (SPRS = 0), symptomatic patients (SPRS > 0), and HC, dcnc values in asymptomatic patients did not differ significantly from those of symptomatic patients but were significantly higher than those of HC (Figure S3), indicating that dcnc alterations are present even in the absence of evident clinical manifestations.

Overall, these results suggest that dcnc is independent of gender, mutation type, age at onset, SPRS disease score, and progression rate, but it is sensitive enough to detect subclinical cellular changes associated with SPG4 pathogenic variants early in the disease progression.

### 3.3 The Dcnc‐Based Method Shows a High Discriminatory Performance for SPG4‐HSP

**FIGURE 3:**
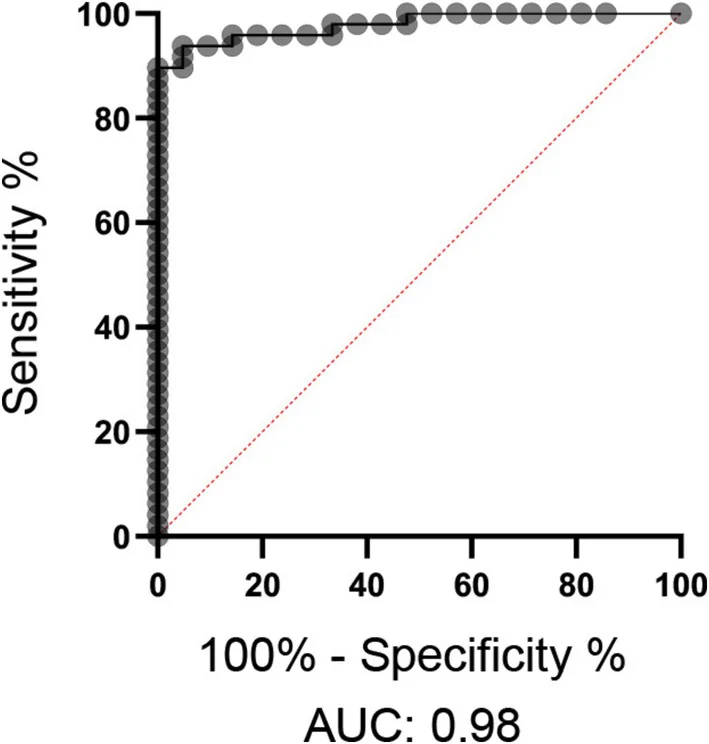
Cross validated classification performance of the dcnc. ROC curve analysis for dcnc (SPG4, *n* = 48; HC, *n* = 21) with 95% confidence intervals. The diagonal red line represents the line of no discrimination (AUC = 0.5).

To assess the diagnostic performance of the dcnc‐based method, a ROC curve was generated using dcnc median values from the full cohort of 48 *SPG4* cohort and 21 HC. The analysis showed an AUC of 0.98 (95% CI 0.95 to 1; *p* < 0.0001; Figure 3), indicating high sensitivity and specificity in distinguishing SPG4‐HSP from controls.

These results reveal that the dcnc is a reliable and mutation‐independent candidate biomarker for SPG4‐HSP. It is capable of distinguishing patient cells from healthy controls, indicating strong discriminatory performance within this cohort regardless of clinical severity.

### 3.4 The Dcnc Reflects Intracellular Spastin Dosage Independently of the Clinical Phenotype

**FIGURE 4:**
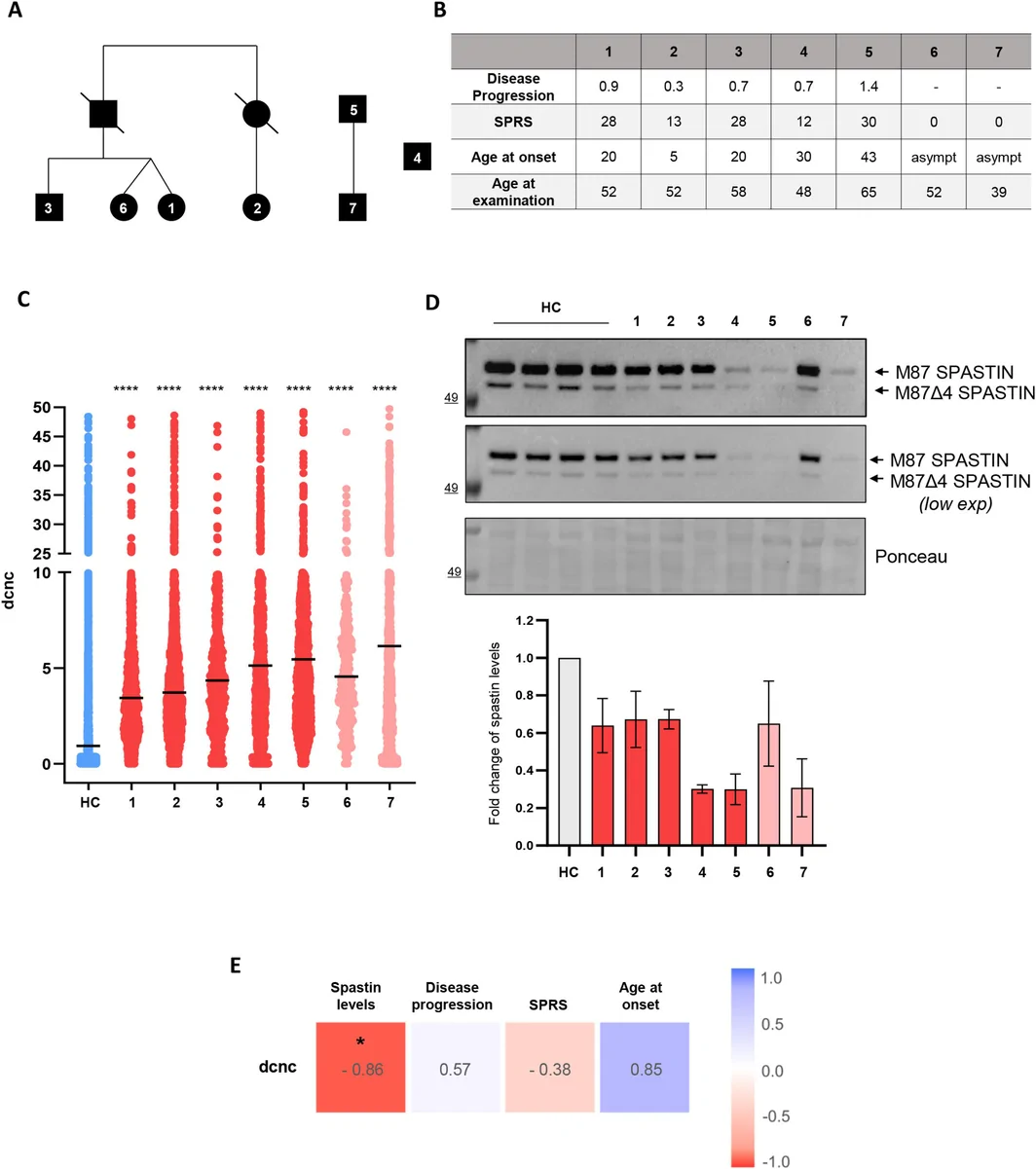
Dcnc and spastin levels in patients carrying the same *SPG4* pathogenic variant. (A) Pedigree of the families carrying c.1215_1219delTATA *SPG4* mutation. All patients are heterozygous for the variant. Circles indicate female family members; squares indicate male family members; filled symbols represent mutation carriers. (B) Clinical characteristics of the patients. (C) Dot plot showing the dcnc values. Each dot represents a single cell and the horizontal black lines indicate the median value. At least 500 cells from each patient sample were analyzed. HC values represent an independent subset of healthy individuals (*n* = 4) analyzed in parallel with patient samples. ****p < 0.0001; *t*‐test, versus HC. (D) Western blot showing spastin protein levels. Spastin levels were normalized to ponceau staining and expressed relative to the mean of HC samples. All samples were processed simultaneously under identical experimental conditions, minimizing potential technical variability. Quantification is reported as the mean ± SD of three technical replicates. (E) Correlation analyses between dcnc median values and indicated clinical data or spastin protein levels. Heatmap shows Pearson's correlation, color coded as follows: Blue (positive), white (none) and red (negative). *p < 0.05.

To assess whether dcnc can detect differences among individuals carrying the same *SPG4* mutation, we analyzed seven heterozygous carriers of the c.1215_1219delTATA variant from three unrelated families, including two asymptomatic carriers (Figure 4). Despite sharing the same genotype, carriers exhibited marked clinical heterogeneity, with SPRS scores ranging from 0 to 30 (mean ± SD: 16 ± 13), age at onset 24 ± 14 years, and disease progression of 0.8 ± 0.4 SPRS points/year.

Consistent with previous observations [22] and (Figures 1A and S3), PBMCs from all carriers displayed elevated dcnc values compared with HC, confirming cytoskeletal disorganization (Figure 4A–C). Notably, substantial inter‐individual variability in dcnc values was observed among carriers of the same variant. Dcnc values did not correlate with clinical parameters, including SPRS score (Pearson *r* = −0.38, *p* = 0.40), progression rate (*r* = 0.57, *p* = 0.31), or AO (*r* = 0.85, *p* = 0.07; Figure 4E), supporting that dcnc reflects cellular alterations rather than clinical disease burden.

Because the assay is sensitive to spastin‐enhancing interventions [22, 23], we next assessed whether dcnc correlates with endogenous spastin levels. Western blot analysis revealed variable spastin expression across carriers (Figure 4D). Strikingly, dcnc showed an inverse correlation with spastin abundance (Pearson *r* = −0.86, *p* < 0.05; Figure 4E), whereby individuals with lower spastin levels displayed higher dcnc values, indicative of more pronounced cytoskeletal disorganization. Similar data were observed in a pair of individuals carrying the c.1728+1G>A mutation, supporting the inverse relationship between spastin levels and dcnc (Figure S4).

Collectively, these results indicate that dcnc identifies cellular phenotypes associated with *SPG4* mutations independently of clinical manifestations. Moreover, the strong correlation with spastin protein levels strongly supports its utility as a target‐engagement biomarker for emerging spastin‐enhancing therapeutic strategies.

## 4 Discussion

The dcnc‐based method reliably discriminates SPG4‐HSP patients from HC. Among the cellular parameters analyzed, dcnc showed the strongest discriminatory power, outperforming other parameters such as LD number and tubulin intensity.

In addition to dcnc, we observed an increased number of LD in SPG4‐HSP cells compared to controls, consistent with previous findings in zebrafish and cellular models [8, 9]. This supports a link between spastin dysfunction and altered lipid metabolism, suggesting that lipid‐related pathways could be further explored as complementary potential biomarkers for SPG4‐HSP.

Dcnc alterations are detectable not only in symptomatic SPG4‐HSP patients but also in asymptomatic non‐manifesting carriers. Dcnc values in these individuals were significantly higher than those of HC and not substantially different from those of symptomatic patients, indicating that this cellular phenotype precedes the onset of clinical manifestations. These findings suggest that dcnc reflects early, subclinical cytoskeletal alterations directly linked to *SPG4* pathogenic variants.

No significant correlation was found between dcnc and clinical features such as disease severity measured by SPRS, AO, progression rate, mutation type, or gender within our cohort. Although this excludes the utility of dcnc as a prognostic or monitoring tool, it is important to consider this finding within the context of the marked intra‐individual and intra‐familial heterogeneity of SPG4‐HSP. This finding highlights that dcnc captures a core cellular phenotype of spastin dysfunction that is independent of the wide clinical and molecular heterogeneity of SPG4‐HSP. This characteristic makes dcnc particularly valuable in experimental and therapeutic contexts, where mutation‐specific and clinical variability often complicates biomarker readout.

The dcnc phenotype appears specifically associated with *SPG4* among different HSP subtypes [22] and no comparable increase was observed in the other movement disorders analyzed (Figure S5A). Previous studies have shown that dcnc alterations can be induced in HC cells by spastin silencing or inhibition [22] (Figure S5B) and that these alterations are reversible upon spastin‐enhancing treatments in *SPG4* cells [22, 23]. These findings support the use of dcnc as a functional readout of spastin activity, suitable for evaluating target engagement and treatment efficacy in patient‐derived cells. In this regard, dcnc could serve as a non‐invasive biomarker for future proof‐of‐concept studies and early‐phase clinical trials evaluating spastin‐enhancing therapies or other disease‐modifying approaches in SPG4‐HSP.

Importantly, the familial subset analyzed in this study carried a protein‐truncating *SPG4* variant (c.1215_1219delTATA) that we previously demonstrated to cause a ≥ 50% reduction of spastin protein [22], consistent with degradation of the mutant transcript through nonsense‐mediated decay. This provides a model of haploinsufficiency, in which the inverse correlation between dcnc and spastin abundance and the absence of correlation with clinical severity anchors dcnc to the primary molecular consequences of SPG4 haploinsufficiency. These findings strengthen dcnc as a potential target‐engagement biomarker, particularly suited to therapeutic strategies aimed at restoring spastin levels.

The variability in spastin levels observed among individuals carrying the same mutation is consistent with the known intrafamilial heterogeneity of SPG4‐HSP, suggesting that such variability may represent a broader feature of the disease and supporting the use of dcnc as a functional readout of spastin dosage. Further investigation of the sources and extent of inter‐individual variability across SPG4 variants will be important.

Another important direction for future research is the integration of dcnc with complementary biomarkers addressing other aspects of SPG4‐HSP pathology. Among fluid biomarkers, sNfL has emerged as a sensitive indicator of axonal injury. Moderately increased sNfL levels in *SPG4* patients compared with controls have been reported, although without correlation with disease severity or progression [16]. These findings suggest that sNfL may reflect subclinical neuronal injury rather than clinical decline, and could therefore complement dcnc, which captures spastin‐related cytoskeletal dysfunction in peripheral cells. Reduced levels of acetylated α‐tubulin in PBMCs of SPG4‐HSP patients have also been reported [26]. Likewise, advanced neuroimaging techniques, including MRI‐based quantitative assessment, both in the brain and spinal cord, offer structural insights into upper motor neuron degeneration and may provide additional sensitivity in detecting disease‐related changes [27, 28]. A multimodal biomarker strategy combining dcnc (cellular dysfunction), sNfL (axonal injury), and neuroimaging (structural degeneration) could therefore improve patient stratification, facilitate early detection of subclinical pathology, and provide robust tools for monitoring target‐engagement and treatment response in clinical trials.

### 4.1 Study Limitation and Future Perspectives

The strength of our findings is supported by the relatively large and clinically diverse cohort analyzed, encompassing 48 patients with distinct *SPG4* variants, a broad clinical spectrum—from asymptomatic carriers to severely affected individuals—and multiple recruitment centers. This enhances the generalizability of our conclusions and reflects real‐world variability in *SPG4* populations. The multicentre recruitment was feasible since we had previously demonstrated that the assay provides comparable results when analyzing PBMCs under pre‐specified conditions (e.g., isolated from whole blood maintained at room temperature for 48 or 72 h or PBMCs frozen in DMSO and thawed, analyzed upon 60 h of PHA treatment to promote cell cycle entry, 22 and Figure S5C), and in the present study no significant differences were observed among the three participating centres.

Nonetheless, some subgroup analyses, particularly those comparing extreme clinical phenotypes, were limited by smaller sample sizes, potentially reducing the power to detect subtle correlations between dcnc and clinical measures. Our study design was cross‐sectional, preventing assessment of whether dcnc values change longitudinally with disease progression. Future longitudinal and interventional studies will be essential to determine the temporal dynamics and responsiveness of dcnc to therapeutic modulation. Additional work should also explore its relationship with other emerging biomarkers, including neuroimaging and fluid‐based measures, to better define its role within a broader biomarker framework. Correlation studies integrating dcnc with neuroimaging or neuro‐related molecular markers could also clarify its relationship with neuronal dysfunction in vivo.

Overall, this study supports the dcnc‐based assay as a robust, non‐invasive, and mutation‐independent peripheral cell‐imaging potential biomarker for genetically and clinically diverse SPG4‐HSP.

## Author Contributions

G.F.: investigation, validation, visualization, data curation. V.L.: methodology, data analysis, visualization, resources, funding acquisition, writing – review and editing. G.D.Z.: investigation, data curation. F.D.C.: investigation. data curation. M.B.: investigation. data curation. N.S.: investigation. data curation. A.F.: investigation. data curation. S.R.: resources, writing – review and editing. F.M.S.: resources, writing – review and editing. G.S.: resources writing – review and editing, investigation. C.C.: conceptualization, funding acquisition, writing – review and editing, resources. F.S.: funding acquisition, writing – original draft, methodology, visualization, writing – review and editing, data curation, supervision. C.R.: conceptualization, funding acquisition, methodology, writing – original draft, writing – review and editing, data curation, project administration, supervision. All the authors read and approved the final manuscript.

## Funding

This study was supported by grants from Telethon #GMR23T2157, AFM‐Telethon #24904, Euro‐HSP Federation, and CNR (DSB.AD006.371‐InvAt‐FOE2022) to CR; the European Union‐Next‐Generation EU within the MUR National Recovery and Resilience Plan‐M4C2‐Action 1.1, “PRIN 2022” (#2022WYCLTR) to CR and CC; Tom Wahlig and HSP‐Selbsthilf Foundations to FS; Telethon # GJC21131 to FMS and GS Ricerca Corrente 2025–2026 to FMS and MB. The funders had no role in study design, data collection and analysis, decision to publish, or preparation of the manuscript.

## Conflicts of Interest

The authors declare no conflicts of interest.

## Supporting information


**File S1:** Supporting Information.

**Figure S1:** Cohort composition and representative PBMC images.

**Figure S2:** Individual‐level distribution of single‐cell values per parameter.

**Figure S3:** Dcnc analysis of symptomatic and asymptomatic SPG4 carriers compared to HC.

**Figure S4:** Relationship between Dcnc analysis and spastin protein levels in additional SPG4 carriers.

**Figure S5:** Dcnc measurements in additional experimental settings.

## Data Availability

The data that support the findings of this study are available from the corresponding author upon reasonable request.
